# Expressive Vocabulary Growth After Pediatric Auditory Brainstem Implantation in Two Cases' Spontaneous Productions: A Comparison With Children With Cochlear Implants and Typical Hearing

**DOI:** 10.3389/fped.2019.00191

**Published:** 2019-05-09

**Authors:** Jolien Faes, Steven Gillis

**Affiliations:** CLiPS, Department of Linguistics, University of Antwerp, Antwerp, Belgium

**Keywords:** auditory brainstem implantation, pediatric, oral language, vocabulary growth, cumulative vocabulary

## Abstract

Auditory brainstem implants (ABI) are recently being used to restore hearing of children with a congenital hearing loss, due to for instance the absence of auditory nerves. Thus far, the literature has focused on perceptual outcomes. The present study is among the first ones to investigate the spoken language development after implantation. The lexical development of children with ABI is examined longitudinally in comparison to children with typical hearing and children with cochlear implants. Results show that children with ABI still have smaller spoken vocabularies as compared to (hearing) age-matched children with cochlear implants and children with typical hearing. Implications will be discussed.

## Introduction

This study examines the lexical development of congenitally deaf children with auditory brainstem implants (ABI), in comparison to children without hearing loss, viz. children with normal hearing (NH), and another group of congenitally deaf children, viz. children with cochlear implants (CI).

Auditory brainstem implantation (ABI) is a very recent technique in pediatric hearing restoration, applied in children with a severe-to-profound hearing loss who do not benefit from cochlear implantation. A cochlear implant bypasses a deficit in the cochlea by inserting an electrode array (into the cochlea itself) that stimulates the auditory nerve. But, in children with malformed cochlea or absent auditory nerves, cochlear implantation will not lead to the desired outcomes. Since the beginnings of this century, these children are candidates for auditory brainstem implantation. In ABI an electrode array is inserted directly onto the cochlear nucleus of the brainstem, bypassing the cochlea and the auditory nerves (1).

## Background

Children with ABI make clear progress in sound perception with longer device use. In the opinion of Sennaroglu et al. (2), most children with ABIs obtain auditory thresholds between 30 and 60 dB HL. Please note that this simply reflects sound detection. Children with ABI are able to discriminate and identify sounds and phonetic contrasts (e.g., /m-s/) (3, 4). In a study of 35 children with ABI implanted between 1 and 5.5 years of age, 71% of the children reached CAP scores of at least 5 (on a 8 point scale), indicating that they could understand simple phrases without lip reading (5, 6). Four out of 35 children reached a CAP score of six, indicating that they could also understand conversatisons without lip reading; two children reached a CAP score of seven, indicating that they use the telephone with a familiar person, and one child even reached the highest score of eight and was able to use the telephone also with unfamiliar persons.

Few studies investigated the speech and language production after ABI in children and adolescents (1, 3, 4, 7–10). Most of these studies reported very general results–the presence of vocalizations, words, and sentences is mentioned–without providing any further details about these aspects. In two case studies, Faes and Gillis (10) showed that young children with ABI started to use spoken words after two to 3 years of device use. Eisenberg et al. (8) investigated the children's speech productions in more detail. They concluded that children with ABI start to use basic word patterns and vary from 0 to 100% accuracy in phoneme production. Eisenberg et al. (8) used a picture-naming task in their study, leaving the question unanswered if, which and how many words children use in daily life. To the best of our knowledge, little to no information is available on the spontaneous word use of young children with ABI.

### Lexical Development

The ability to learn words and to build up a vocabulary is crucial for language acquisition. Typically developing children exhibit a fast pace of lexical development in the second year of life and numerous studies examined the developmental trends and the pace of vocabulary increase (see e.g., (11) for an overview). Lexical development is the driving force of several other aspects of children's language development. First, lexical development is a key factor for phonological and grammatical development. For instance Ainsworth et al. (12) showed that phonological representations become more accurate with increasing vocabulary size. Moreover, different aspects of phonological development are related more to vocabulary size than to children's chronological age (13–16), and also grammatical skills are linked to early lexical development (17). Second, early expressive vocabulary development is shown to predict later vocabulary development (18, 19), reading comprehension and literacy (20–22) academic skills and executive functioning (23).

### Children With Hearing Loss

Cochlear implantation has been applied for several decades already and these children's language development after implantation has been studied extensively. Several studies have shown that lexical skills are among the best-developed language skills in children with CI after implantation [e.g., (23–28)]. Some studies even showed that children with CI's lexical development is similar to that of age-matched hearing peers after several years of device use (24, 27, 28).

For children with ABI, there is little or no information about their lexical development. At present it remains even unknown if they are able to develop spoken vocabulary and use words in spontaneous speech. Moreover the question is whether their lexicon develops in a comparable way to that of children with CI, or even NH. The present paper aims to investigate the lexical development of children with ABI in spontaneous speech and compare it to that of children with NH and CI.

## Methods

### Participants

The participating children with implants (ABI or CI) all had a congenital bilateral sensorineural hearing loss of more than 90 dB HL. Inclusion criteria for this study were: children had to be raised in Dutch, completed at least 1 year of follow-up, and no other health or developmental issues were reported.

Since 2015, only eight children received an ABI in Belgium. Two children (S1 and S2) with ABI implantation matched the inclusion criteria listed above. These criteria limit the pool of participants, especially for the children with ABI. The criterion “Dutch speaking” limits the geographic area to only the northern part of Belgium. When limiting our sample to only children without additional developmental or health problems [in line with Eisenberg et al. (8)], three children with ABI that could have been included. One child did not meet the 1-year follow-up criterion, and was excluded as well. The hearing loss of the two children with ABI was due to the absence of the auditory nerves. Pure tone average (PTA) hearing thresholds before implantation were 120 and 116 dB HL. The children were implanted around their second birthday (24 and 25 months of age, Med-El Synchrony and Med-El Concerto, respectively). In both children, nine out of 12 electrodes could be fitted. Two years after implantation, PTA thresholds had improved to 37.5 and 43 dB HL CAP score for S1 was three after 2 years of device use, no data were available for S2. S1 was followed 15 months, starting 1 year after implantation. S2 was followed 18 months, starting 23 months after implantation. First words appeared already in the earliest data files. Both children used oral language, largely supported by Flemish Sign Language.

Nine children (S3–S11, Table 1) received cochlear implants (CI) (Nucleus-24). The mean PTA before implantation was 112.56 dB HL (*SD* = 9.13). The mean age at implantation was 11.92 months (*SD* = 5.25). At 2 years of age, the mean PTA had improved to 39.78 dB HL (*SD* = 8.67). The mean CAP score was 6 after 2 years of device use (range 5–7, see Table 1). All children were followed from implantation up to 30 months after implantation. Data are included from the appearance of first word productions (Table 1). All children were raised in orally, supported with a limited amount of lexical signs.

**Table 1:** Characteristics of the children with CI.

**ID**	**PTA unaided**	**PTA CI (24 months)**	**Cap sore (24 months of CI use)**	**Age 1st CI**	**Age activation 1e CI**	**Hearing age First word use**	**Hearing age End of the study**	**Chronological age First word use**	**Chronological age End of the study**	**Age 2nd CI**
S3	120	48	6	13.49	14.89	5	24	21	39	–
S4	120	30	7	6.69	7.66	8	28	16	36	–
S5	115	33	6	10.00	11.66	8	26	20	38	–
S6	113	48	7	18.16	19.34	1	29	20	44	–
S7	93	38	6	16.89	17.89	0	24	18	42	–
S8	120	53	5	8.76	9.66	6	26	16	36	–
S9	117	42	7	5.16	6.13	9	30	15	36	15.00
S10	112	38	6	19.46	21.13	7	25	23	46	–
S11	103	28	6	8.69	9.69	5	26	15	36	23.00
*Mean*	*112.56*	*39.78*	*6.22*	*11.92*	*13.12*	*5.44*	*26.44*	*18.22*	*39.22*	*52.50*
*SD*	*9.13*	*8.67*	*0.67*	*5.25*	*5.40*	*3.13*	*2.13*	*2.91*	*3.87*	*27.03*

*PTA levels are presented in dB HL*.

*Ages are represented in months*.

Finally, 30 children with NH (S12–S41) were followed between 6 and 24 months of age. For each child, data were included from the appearance of their first words. The mean age at first word production was 13.27 months (*SD* = 1.74). Individual data are shown in Table 2.

**Table 2:** Characteristics of children with NH.

**ID**	**Age first words (months)**
S12	14
S13	12
S14	14
S15	13
S16	11
S17	15
S18	16
S19	12
S20	15
S21	14
S22	16
S23	16
S24	14
S25	12
S26	15
S27	11
S28	12
S29	12
S30	13
S31	12
S32	14
S33	13
S34	12
S35	10
S36	12
S37	17
S38	13
S39	11
S40	14
S41	13
*Mean*	*13.27*
*SD*	*1.74*

The Ethical Committee for the Social Sciences and Humanities of the University of Antwerp approved this study and all parents signed an informed consent form.

### Data Collection and Transcription

For all corpora, data collection consisted of monthly 1-h video-recordings at the child's home and involved spontaneous, unstructured interactions between the child and caregiver(s). Parents were told to act “as they normally did” and none of the settings were not standardized by providing specific toys or pictures. The video-recordings were transcribed in CHILDES'CLAN according to the CHAT conventions (29). The full 1-h video recordings of the ABI corpus were transcribed, given the limitedness of the sample. For the CI and NH corpora, a 20-min selection of each 1-h video was made in order to keep transcription time within reasonable limits. Only complete interactions were included and for instance very noisy passages and silent passages (e.g., when the child was eating) were excluded (30, 31). The time-investment for data collection and transcription averaged 10.5 h (30).

Each utterance was identified and the speaker was labeled. Vegetative and pure (dis-)comfort sounds, e.g., laughing, were excluded. Children's oral productions were transcribed as either lexical or prelexical, following the procedure of Vihman and McCune (32). Lexical productions equal words, prelexical productions equal all precursors to words, i.e., canonical babble and other types of vocalizations. Lexical productions were transcribed orthographically. A second transcriber checked 5% of the transcriptions. The percentage of agreement on items classified as lexical or prelexical was 85.26%.

### Data Analyses

Lexical development is examined as the cumulative vocabulary size relative to the children's hearing age and children's chronological age. Hearing age is defined as the number of months after the device switch on for the children with ABI and CI. In children with NH, hearing age equals their chronological age. For children with ABI, the hearing ages were between 12 and 27 months for S1 and between 23 and 41 months for S2. For both children, at least a few words appeared already in the first data file. For the children with CI, the mean hearing age at first word production was 5.44 months (*SD* = 3.13) and the mean hearing age at the end of the study was 26.45 months (*SD* = 2.13) (Table 1). For the children with NH, the mean (hearing) age at the start of word use was 13.27 (*SD* = 1.74) and all children had 24 months of (hearing) age at the end of the study (Table 2).

Cumulative vocabulary is a standard measure to estimate children's vocabulary growth in longitudinal, spontaneous data [e.g., (18, 33, 34)]. It is determined by counting the number of new words in each consecutive data file. So, in a first file, i.e., at the youngest hearing age, the number of distinct word types is counted. In the following file, this original number is augmented with the number of new words types, representing the cumulative vocabulary at that point. For each consecutive file, this procedure is iterated. In the present study, inflected forms were counted as distinct types. Onomatopoeic productions were not included as words. The cumulative vocabulary outcomes provide an assessment of the diversity of each child's lexical richness.

Since the transcriptions were based on video-recordings of different time durations, the vocabulary counts were normalized by implementing a bootstrapping procedure following (35). For each child and for each individual data file, the following procedure was adopted. From each data file, a random sample with replacement of 100 lexical items was drawn. Thereof, the cumulative vocabulary at each point in time (for each data file) was determined as described above. This bootstrapping procedure was then iterated 10,000 times, resulting for each child in 10,000 values of cumulative vocabulary for the first data file, 10,000 values for the second data file and so on. The mean value of cumulative vocabulary at each point in time, and thus for each data file, was considered to be a reliable estimate of the child's actual cumulative vocabulary.

## Results

**Figure 1:**
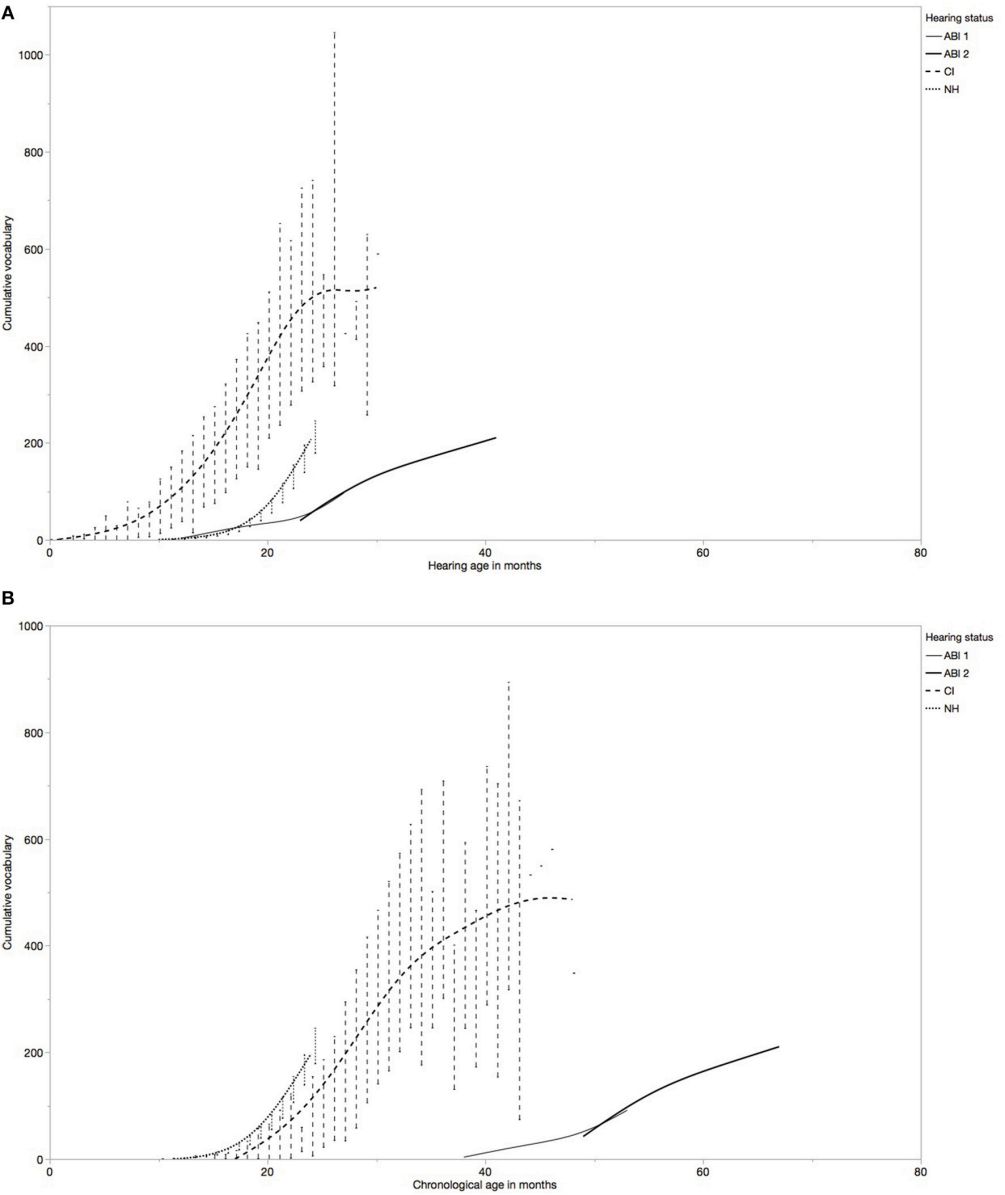
Development of cumulative vocabulary in the three groups of children. **(A)** comparisons on hearing age. **(B)** comparisons on chronological age.

Figures 1A,B show the development of the mean cumulative vocabulary relative to hearing age and chronological age for the children with ABI as compared to the children with CI and NH. There is a clear increase of the cumulative vocabulary in all children.

First, Figures 1A,B show the development of the youngest child with ABI (S1). As compared to children with CI, the cumulative vocabulary of S1 is lower at each point in time for both hearing age and chronological age and the developmental increase seems slower as well. When comparing S1 to children with NH, Figure 1A shows no initial difference between 12 and 17 months of hearing age, but note that the difference in chronological age is ~2 years (Figure 1B). However, the development is much steeper in children with NH than in S1, resulting in lower cumulative vocabulary sizes for the child with ABI as compared to children with NH at the older hearing ages. This difference seems to enlarge with hearing age. No direct comparisons with children with NH with similar chronological ages could be made in this respect.

Figures 1A,B also show the development of the other child with ABI (S2). The cumulative vocabulary of S2 increases steadily over time. Nevertheless, it remains lower than that of the children with CI and NH the entire studied period. Since there was no overlap in chronological age between S2 and the children with NH, no comparisons can be made in this respect. In addition, the developmental increase seems slower for S2 as compared to children with CI and NH. In other words, the initial difference seems to enlarge with increasing hearing and chronological age.

Children with NH's cumulative vocabulary is lower than that of children with CI with similar hearing ages as well. But, Figure 1A shows a faster increase with hearing age for children with NH. In addition, there is an important difference in chronological age between children with CI and NH. When comparing the two groups of children on chronological age in Figure 1B, the children with NH seem to have slightly larger vocabulary sizes as compared to the children with CI. This observation was not tested statistically.

## Discussion

Results showed that children with ABI use words in ordinary conversations and that there is a growth in spoken vocabulary sizes with increasing (hearing) age. This straightforward conclusion has important implications, since lexical development is closely related to phonological and morphological development [e.g., (13–17)]. Early lexical skills and expressive vocabulary predict later vocabulary and other language outcomes, reading and literacy, executive functioning, and even academic achievement in children with NH and CI [e.g., (18–21)]. In future studies, it is important to examine if the early vocabulary skills of children with ABI can be related to other aspects of their (language) development as well. In any case, the results can be interpreted optimistically in this respect.

A comparison of the two children with ABI to children with CI (*N* = 9) and NH (*N* = 30) reveals differences in the precise vocabulary sizes at similar hearing ages and similar chronological ages. Children with ABI's cumulative vocabulary sizes are lower than those of children with CI and those of children with NH and the differences seem to enlarge over time. Moreover, their vocabulary sizes fall outside of the 95% confidence intervals of the two other groups of children, irrespectively of the measure of comparison.

## Concluding Remarks

In this paper the groups of children were compared relative to their hearing age in order to avoid methodological issues of different onsets of hearing. Hearing age has been used extensively in the literature on children with CI [for instance (25, 36–38)]. But, hearing age is still correlated to chronological age. In our sample, the children with ABI's chronological ages varied between 3 and 6 years, whereas the chronological age of children with NH varied between 6 months and 2 years. This implies that at a hearing age of, say, 12 months, the children with NH in our sample are 1 year old (chronological age) while the children with ABI are 3 years old. Thus, at similar hearing ages, the children's chronological ages differ drastically. Similarly, the mean implant age of children with CI was 1 year lower than that of children with ABI, resulting in a mean chronological age difference of 1 year.

These considerations are important to interpret the results. Initially, between 12 and 17 months of hearing age, the child with ABI (S1) and children with NH have similar cumulative vocabulary sizes. Later on, the differences seem to become apparent and seem to enlarge. Reasonably, this initial similarity is due to the use of hearing age as a measure of comparison. The cognitive, motor and physical development of 1-year old and 3-year old children differ tremendously (39). The children with NH just started to use words at 12 months of hearing age, whereas S1 already has some lexical knowledge on the basis of sign language use earlier in life. Moreover, the motor control at 3 years of age is developed to a larger extent than that of a 1-year-old. These considerations are also attested when comparing the groups of children on chronological age.

Figure 1A seem to indicate that cumulative vocabulary size is larger in children with CI than in children with NH. But, similarly, this can be explained by the interference of chronological age differences. The mean hearing age at first word production was 13.27 months for children with NH and 5.44 months for children with CI. But this means that the children with NH were about 13 months old, whereas the children with CI were about 17 months old. As children with NH grow older, the increase of cumulative vocabulary size seems much steeper than that of children with CI. Once again, this is confirmed when comparing the groups on chronological age. At similar chronological ages, children with NH have slightly larger vocabulary sizes.

Children with ABI already used lexical signs in their daily communication before implantation. Our results showed that it takes about 1 year of device use before the first words appear in their spoken language as well. It is striking that the lexical knowledge of sign language does not lead to an earlier shift of word use in spoken language. Moreover, the children with CI are using spoken words much earlier (5 months of hearing age), even though the children with ABI are older and thus should have more developed cognitive skills. There are several explanations. A first factor might be the age at implantation. The children with CI are implanted around their first birthday, whereas the children with ABI received their implant at age two. Earlier implantation has beneficial effects on vocabulary development for children with CI (40, 41), so it may be that the later age at implantation contributes to the later spoken word use of the children with ABI.

It may also be that there are striking differences between the implant children use (ABI vs. CI) and the perceptual benefit they have from it, but this route is still entirely open for research. Probably, an important factor is the amount of sign language use. As they are implanted at a later age, children with ABI use much more sign language than the children with CI. It is plausible that they are more proficient in sign language than children with CI. But this proficiency may impede spoken word use. Studies showed that children with CI implanted earlier in life use more often an oral communication mode, are more likely to shift from sign to oral language use and that this sign-to-oral shift appears earlier in life than in children with CI implanted later (42). And, children with CI relying less on sign language show language advantages, including effects on vocabulary (27, 43). It is very likely that similar effects are apparent in children with ABI.

One limitation of the present paper is the number of participants in the groups with hearing loss. Since pediatric ABI implantation is a recent innovation, the number of potential participants is still very limited. Another limitation of the study is that we have not measured vocabulary size in children with profound deafness who do not use any sensory aids, and therefore we have not estimated any possible changes in vocabulary size that might happen even in the absence of auditory input. But the results remain important on different levels. From a theoretical point of view, our results show that the brain is able to use the electric stimulation of the brainstem implant in a functional way. When weighing the surgical risks to the benefits of ABI implantation, the present results should be taken into account.

Clinically, our results can be a basis in speech and language therapy for children with ABI. Thus far, language therapy for these children is entirely based on that for children with CI, without (m)any detailed linguistic investigations of the comparability of these children. The present paper is among the first ones to do so. The results show that children with ABI enlarge their spoken vocabulary sizes with age and increasing implant use. We suspect that this increase in vocabulary size is greater than it would have been if the children had not been provided with ABIs. Nevertheless, there is still a gap between these children and children with CI. In clinical follow-up, focus on lexical development may be crucial in order to enlarge their spoken vocabulary sizes which may prove beneficial for other aspects of language development.

To conclude, children with ABI develop spoken language skills. Their word use increases steadily with longer ABI experience. Even though there is still a difference as compared to hearing age-matched children with CI and NH, the results are promising for children with ABI's spoken language development.

## Ethics Statement

This study was carried out in accordance with the recommendations of the Ethical Committee for the Social Sciences and Humanities of the University of Antwerp (SHW_17_16) with written informed consent from all subjects. The parent of all subjects gave written informed consent in accordance with the Declaration of Helsinki. The protocol was approved by the Ethical Committee for the Social Sciences and Humanities of the University of Antwerp

## Author Contributions

JF and SG contributed conception and design of the study. JF organized the database. JF and SG performed the analysis together. JF wrote the first draft of the manuscript. JF and SG wrote sections of the manuscript. All authors contributed to manuscript revision, read, and approved the submitted version.

### Conflict of Interest Statement

The authors declare that the research was conducted in the absence of any commercial or financial relationships that could be construed as a potential conflict of interest.
